# Pulsed Electric Field Technology for the Extraction of Glutathione from *Saccharomyces cerevisiae*

**DOI:** 10.3390/foods13121916

**Published:** 2024-06-18

**Authors:** Alejandro Berzosa, Javier Marín-Sánchez, Ignacio Álvarez, Cristina Sánchez-Gimeno, Javier Raso

**Affiliations:** Food Technology, Facultad de Veterinaria, Instituto Agroalimentario de Aragón-IA2, Universidad de Zaragoza-CITA, 50013 Zaragoza, Spain; aberzosa@unizar.es (A.B.); j.marin@unizar.es (J.M.-S.); ialvalan@unizar.es (I.Á.); anacris@unizar.es (C.S.-G.)

**Keywords:** glutathione, pulsed electric fields, extraction, antioxidant, *Saccharomyces cerevisiae*

## Abstract

Glutathione is a potent antioxidant that has shown promise in enhancing the processing of various foods and drinks such as bread and wine. *Saccharomyces cerevisiae* stands as a primary microorganism for glutathione production. This study sought to assess the potential of pulsed electric fields (PEFs) in extracting glutathione from *S. cerevisiae* cells. Yeast cells were subjected to PEF treatment (12 kV/cm, 150 µs) followed by incubation at varying pH values (4.0, 6.0, and 8.0) and temperatures (4 °C and 25 °C). Glutathione and protein extraction were assessed at different incubation times. Within one hour of incubation, PEF-treated yeast cells released over 60% of their total glutathione content, irrespective of pH and temperature. Notably, the antioxidant activity of the resulting extract surpassed that obtained through complete mechanical cell destruction and hot water, which form the conventional industrial extraction method in the glutathione industry. These results suggest that PEF could offer a rapid and more selective procedure, improving the extraction of this bioactive compound.

## 1. Introduction

Glutathione, a tripeptide composed of L-glutamate, L-cysteine, and glycine, is a vital compound with a low molecular weight of 307.3 g/mol. It is the most abundant non-protein thiol in living organisms, including eukaryotic cells. While more than 90% of glutathione exists in its reduced form (GSH), oxidative processes can convert it to its oxidized form (GSSG), or it may bind to other molecules through glutathionylation [1]. Glutathione holds a crucial role in cellular functions and human health by serving as one of the most important antioxidants. It protects cells from oxidative damage, maintains redox homeostasis, enhances metabolic detoxification, and regulates immune system function [2]. Furthermore, the ratio of GSH to GSSG has been explored as a potential therapeutic biomarker and treatment target in various chronic and age-related diseases [3]. Glutathione is primarily sourced through two mechanisms: endogenous synthesis within cells and exogenous intake via dietary sources. While certain foods, particularly fruits and vegetables, contain varying levels of glutathione that can stimulate intracellular GSH synthesis [4], supplementation with GSH may be considered during periods of increased oxidative stress [5,6].

Beyond its physiological significance, glutathione has attracted significant attention in the food industry for its potent antioxidant properties, ability to modify texture, and role in aroma enhancement. These attributes contribute to the preservation of food quality and extension of shelf life [7,8,9]. Particularly in the oenological sector, glutathione has emerged as a prominent alternative to the use of sulfur dioxide due to its antioxidative attributes [10,11,12,13].

The growing demand for glutathione across various industrial sectors, including pharmaceuticals, cosmetics, and food, has spurred continuous advancements in production, extraction, and purification techniques and methodologies. While some research has focused on glutathione production and extraction from plant tissues [14], the industrial scalability of these approaches remains limited by cost considerations. As an alternative, extensive research over the past four decades has been dedicated to exploring microorganisms capable of producing glutathione such as *Saccharomyces cerevisiae* or *Candida utilis* [15].

Research and patents have extensively explored strain improvement and process optimization for industrial glutathione production [1,16]. However, there is a notable scarcity of openly published literature addressing the extraction procedures of GSH. While hot water extraction is the classical method employed at the industrial scale, alternative approaches such as ethanol extraction [17] or aqueous two-phase systems [18] have been proposed. The development of innovative techniques that enhance extraction efficiency and facilitate the attainment of a high-purity extract from yeast cells is of significant interest for the commercial exploitation of glutathione.

In recent years, pulsed electric field technology (PEF) has emerged as a promising method for enhancing the extraction of bioactive compounds from microorganisms [19], including glutathione [20,21]. This technology involves subjecting cells to high-intensity electric fields (kV/cm) for brief periods in the form of short pulses (µs), which alters the selective permeability of the cell’s cytoplasmic membrane [22]. This phenomenon, known as electroporation, is characterized by the formation of small pores in the cell membrane, facilitating the release of intracellular components [23]. This non-thermal approach offers several advantages over conventional extraction methods, such as minimizing thermal degradation and enhancing the purity of extracts by maintaining their cellular structure [24].

The aim of this study was to assess the impact of various factors on the extraction of glutathione from *Saccharomyces cerevisiae* biomass treated with PEF in terms of enhancing both the extraction yield and the purity of the extracts.

## 2. Materials and Methods

### 2.1. Strain, Culture Conditions, and Yeast Suspensions

A commercial strain of *S. cerevisiae* SafAleTM S-04 (Fermentis-Lesaffre, Marcq-en-Barœul, France) was used in this investigation. Yeast cultivation was conducted at 25 °C in a 20 L stainless steel barrel containing 12 L of Sabouraud dextrose liquid medium (Oxoid, Basingtok, UK) under agitation with a magnetic stirrer. Yeast growth was monitored by the plate counting method in potato dextrose agar (PDA) (Oxoid, Thermo Fisher Scientific, Waltham, MA, USA) after incubation of plates at 25 °C for 48 h. The experiments were conducted with cells in the stationary growth phase after 48 h of incubation.

Yeast cells in the stationary growth phase were washed twice with distilled water by centrifugation (3000× *g* for 5 min at 20 °C) and were resuspended in citrate–phosphate McIlvaine buffer of 2 mS/cm conductivity and different pH values (4.0, 6.0, or 8.0) to a concentration of 10^9^ UFC/mL, which corresponded to 22.41 ± 3.47 g dry cell weight/L (g d_w_/L).

### 2.2. PEF Processing

Yeast biomass underwent PEF treatment in a continuous-flow chamber utilizing commercial PEF equipment (Vitave, Prague, Czech Republic). This equipment can deliver monopolar square waveform pulses reaching up to 20 kV, with an adjustable pulse width ranging from 500 ns to 100 µs and a maximum current intensity of 500 A.

**Figure 1:**
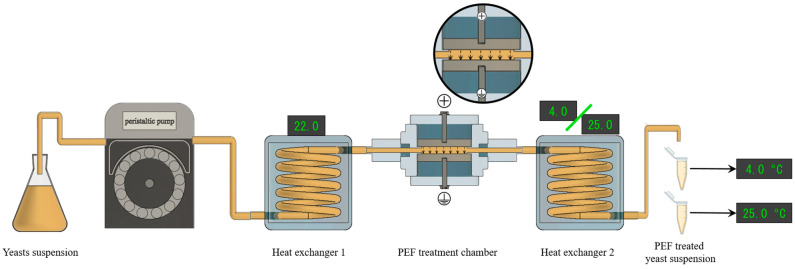
Scheme of continuous-flow PEF treatment system used for this study. The liquid flow is produced from left to right, and after treatment, suspensions were incubated at both 4 and 25 °C.

A peristaltic pump (BVP, Ismatec, Wertheim, Germany) circulated the yeast biomass at a flow rate of 5.10 ± 0.05 L/h through a titanium parallel electrode chamber with dimensions of 0.4 cm gap, 3.0 cm length, and 0.5 cm width. Monopolar square waveform pulses, with a width of 3 µs, were applied at electric field strengths of 12, 15, and 18 kV/cm and frequencies ranging from 15.9 to 119.4 Hz, resulting in total treatment durations of between 20 and 150 µs, calculated by multiplying the total number of pulses applied by the pulse width. These treatments corresponded to total specific energies ranging from 9.6 ± 2.9 to 109.8 ± 5.1 kJ/kg of yeast suspension. The actual voltage during the treatments was monitored using a high-voltage probe (Tektronik, P6015A, Wilsonville, OR, USA) connected to an oscilloscope (Tektronik, TDS 220). The output temperatures, ranging from 24.9 ± 0.6 to 48.7 ± 1.1 °C, were measured using a K-type thermocouple integrated into the circuit (Ahlborn, Holzkirchen, Germany). Before the PEF treatments, the yeast biomass was tempered at 22.0 ± 0.5 °C using a heat exchanger located before the treatment chamber. After PEF treatment, the yeast suspension was then rapidly cooled to the subsequent incubation temperature (4.0 or 25.0 °C) in less than 5 s using a heat exchanger after the treatment chamber (Figure 1).

#### Determination of the Effect of PEF on the Electroporation of *S. cerevisiae*

The evaluation of cytoplasmic membrane permeabilization, resulting from the irreversible electroporation induced by PEF treatment, was conducted using propidium iodide (PI) uptake assessment. PI, a hydrophilic molecule with a low molecular weight (660 Da), can only cross permeabilized cytoplasmic membranes. Within the cytoplasm, PI binds to nucleic acids, forming a fluorescent complex with excitation and emission peaks at 535 nm and 617 nm, respectively. After PEF treatments, 50 μL of PI (0.1 mg/mL) was added to 450 μL of yeast biomass suspension, previously resuspended in phosphate-buffered saline (PBS) (Sigma-Aldrich, Saint Louis, MO, USA) to reach a concentration of 10^8^ CFU/mL, to achieve a final PI concentration of 0.015 mM. Suspensions were then incubated for 10 min at room temperature, as described by Martínez et al. (2016) [25]. Epifluorescence microscopy (Nikon, Mod. L-Kc, Nippon Kogaku KK, Tokyo, Japan) was used for the determination of electroporated yeast cell counts. The results are presented as the percentage of electroporated cells.

### 2.3. Hot Water Treatment

We added 1.0 mL of the yeast suspension to a 1.5 mL Eppendorf tube and heat treated that in an microtube shaking incubator (I-4002-HCS, Labnet, Edison, NJ, USA). The temperature was monitored using a K-type thermocouple inserted into a separate Eppendorf tube with yeast suspension. Once 95 °C was reached in the yeast suspension, the treatment was upheld for 3 min. Then, samples were centrifugated at 1593× *g* for 10 min to obtain the supernatant, which was subsequently analyzed.

### 2.4. Bead Milling Treatment

The total concentration of compounds in yeast cells was determined after total destruction of the cells by mechanical rupture using a bead mill (Mini-Beadbeater-Plus; BioSpec, Bartlesville, OK, USA). In a 2.0 mL screw-capped tube, 1.5 mL of the yeast suspension was combined with glass beads (0.5 mm diameter) at a weight ratio of 1:5 (glass bead/yeast biomass). The progress of mechanical disruption was observed using a microscope (Eclipse E400, Nikon, Tokyo, Japan). Ten cycles, each lasting 70 s with cooling intervals in an ice water bath, were conducted to disintegrate more than 90% of the cells. Following this, the suspensions were centrifuged at 1593× *g* for 10 min to obtain the supernatant, which was subsequently analyzed for the total compound concentration.

### 2.5. Analytical Measurements

#### 2.5.1. Reduced Glutathione Concentration

The quantification of reduced glutathione (GSH) was conducted through a colorimetric method utilizing 5.5′-Dithiobis-(2-nitrobenzoic acid) (DTNB) (Thermo Fisher Scientific), following a modified version of the procedure outlined by Ganeva et al. (2020) [21]. GSH reacts with DTNB to produce the chromophore TNB (5-thio-2-nitrobenzoic acid), with maximum absorbance observed at 412 nm. Briefly, 960 µL of phosphate-buffered saline (PBS) at pH 7.5 containing 5.6 mM EDTA (Sigma-Aldrich) was mixed with 20 µL of a 0.4% DTNB solution in the same buffer, along with an additional 20 µL of the sample. After incubating for 2–10 min at room temperature, absorbance was measured at 412 nm. Glutathione concentrations were determined using a standard curve prepared with reduced L-glutathione (Sigma-Aldrich) ranging from 3.9 to 2000 µg/mL. The results were expressed as milligrams of reduced L-glutathione per gram of dry weight or as the percentage of extraction of the total content inside the cells determined after the total destruction of the cells by bead milling.

#### 2.5.2. Protein Concentration

Protein extraction was assessed using the commercially available Pierce BCA Protein Assay Kit (Thermo Fisher Scientific, Rockford, IL, USA). This assay utilizes the Biuret reaction, where proteins reduce Cu^2+^ to Cu^1+^ in an alkaline environment, followed by the colorimetric detection of the cuprous cation (Cu^1+^) using a single reagent containing bicinchoninic acid (BCA). In summary, 200 µL of the working reagent was mixed with 25 µL of the appropriately diluted sample in distilled water. The mixture was incubated at 37 °C for 30 min, and absorbance was measured at 562 nm. A standard curve was prepared using albumin concentrations ranging from 2.0 to 0.06 mg/mL, and the results were expressed as milligrams of albumin equivalents per gram of dry weight or as the percentage extraction of the total content inside the cells determined after the total destruction of the cells by bead milling.

#### 2.5.3. Enhancement of the Ratio of Glutathione to Proteins (Δ Ratio Glutathione/Proteins)

Since bead milling results in the complete destruction of cells, the glutathione/protein ratio within the extracts obtained through PEF and hot water treatments was normalized relative to the ratio achieved through bead milling (BM) treatment.

The enhancement in the glutathione/protein ratio (Δ ratio) was calculated using the following equation:

(1) $$∆\;ratio\;\frac{glutathione}{proteins}=\frac{ratio\;\frac{glutathione}{proteins}\;(PEF\;or\;HW)}{ratio\;\frac{glutathione}{proteins}\;BM}$$

#### 2.5.4. Antioxidant Capacity

The antioxidant capacity of yeast extracts was assessed using the DPPH (2,2-Diphenyl-1-picrylhydrazyl) radical scavenging assay, characterized by the color change of the DPPH solution from purple to yellow upon reduction. Samples appropriately diluted in distilled water were mixed 1:1 with DPPH (Sigma-Aldrich) diluted in methanol (0.04 g/L). After 30 min of incubation in darkness, absorbance was measured at 516 nm using methanol–water (1:1) as the blank and a DPPH–water (1:1) as the control. A standard curve was prepared with Trolox (0–10 μg/mL) (Sigma-Aldrich) diluted in water. Antioxidant capacity results were expressed as mg of Trolox equivalents per gram of dry weight. DPPH radical inhibition % was calculated using the following equation:

(2) $$DPPH\;radical\;inhibition\;\left(\%\right)=\frac{A_{B}-A_{S}}{A_{B}}\times 100$$

where A_B_ is the absorbance of the blank (methanol + water) and A_S_ is the absorbance of the sample measured at 516 nm.

### 2.6. Dry Weight Determination

The samples were dried until a constant weight was achieved (30 °C, 15 h) using a centrifugal concentrator (miVac DNA-23050-B00, Ipswich, UK) to determine their dry weight.

### 2.7. Electrophoresis (SDS-PAGE)

Protein detection was carried out using 4–15% Mini-PROTEAN^®^ TGX Stain-Free™ Protein Gels (Bio-Rad, Hercules, CA, USA). In short, 15 µL of undiluted sample was mixed with 14.25 µL of Laemmli Sample Buffer (Bio-Rad) and 0.75 µL of 2-Mercaptoethanol (Bio-Rad), followed by heating at 95 °C for 5 min. Subsequently, 20 µL of the mixture was loaded into the gel, and Precision Plus Protein™ Standard (Bio-Rad) was added directly to the gel. SDS-PAGE was conducted using a Mini Protean^®^ 3 Cell (Bio-Rad) connected to a PowerPac™ Basic Power Supply (Bio-Rad). After electrophoresis, protein bands were visualized by exposing the gel to a Gel Doc™ EZ System with a Stain-Free Tray (Bio-Rad). The gel was irradiated at a wavelength of 302 nm for 5 min, initiating a chemical reaction between the indole of tryptophan and the 2,2,2-trichloroethanol (TCE) incorporated into polyacrylamide gels. Consequently, bands corresponding to specific proteins became visible under UV light [26].

### 2.8. Statistical Analysis

The reported results are presented as the mean ± standard deviation derived from three replicates. Statistical analysis involved a one-way analysis of variance (ANOVA), followed by a Tukey test, using GraphPad Software (version 8.4.2, GraphPad Software Inc., San Diego, CA, USA), with statistical significance set at a threshold of *p* ≤ 0.05.

## 3. Results and Discussion

### 3.1. Selection of PEF Treatment Conditions for Electroporation of Yeast Biomass

The cytoplasmic membrane of yeast acts as a barrier, impeding the movement of substances between the cell and its environment. Enhancing the extraction of intracellular compounds through PEF requires the permanent electroporation of the cytoplasmic membrane, enabling the release of compounds such as glutathione. Previous research has identified a relationship between the extraction yield of cytoplasmic compounds and the proportion of yeast cells irreversibly electroporated by PEF [20]. However, beyond a threshold of 90% electroporated cells, further increases do not significantly improve extraction efficiency. Therefore, this part of the study aimed to investigate the influence of PEF processing parameters (electric field strength and treatment time) on the electroporation of *S. cerevisiae* cells, to identify the most suitable treatment for electroporating at least 90% of the yeast population.

**Figure 2:**
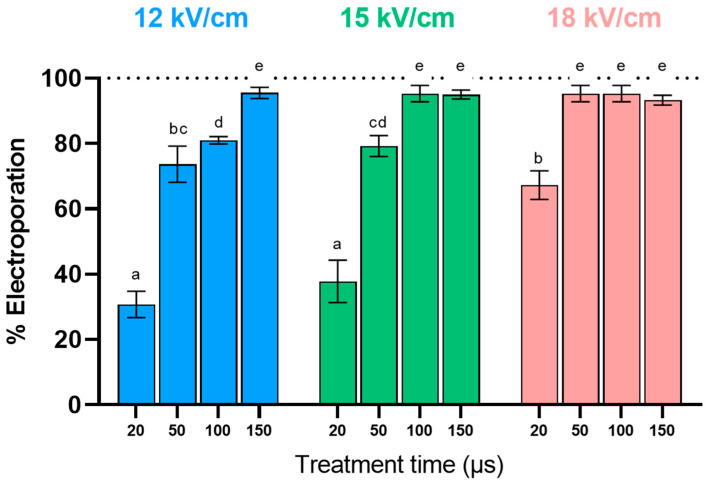
Influence of electric field strength and treatment time on the percentage of electroporated *S. cerevisiae* cells. Different letters denote significant differences (*p* ≤ 0.05).

Figure 2 illustrates the effect of the electric field strength and treatment time on the percentage of electroporated yeast cells. Among the conditions studied, the percentage of permeabilized cells increased with both the treatment time and electric field. While with 20 µs only, about 35% of the population was permeabilized at 12 and 15 kV/cm, the percentage of electroporated cells doubled by increasing the electric field strength to 18 kV/cm. These results align with previous studies indicating that the critical electric field for the manifestation of electroporation in yeast cells is above 10 kV/cm [27,28]. To electroporate over 90% of the population, it was necessary to extend the treatment time to 150, 100, and 50 µs at 12, 15, and 18 kV/cm, respectively. Although the treatment applied at the highest electric field (18 kV/cm) and shortest treatment time (50 µs) corresponded to the treatment with the lowest total specific energy (31.1 ± 2.4 kJ/kg), the selected treatment for further studies was the treatment at the lowest electric field strength (12 kV/cm, 150 µs, 55.3 ±5.1 kJ/kg). From the perspective of implementing the technique at an industrial scale, lower requirements for the electric field result in higher processing capacities without the need for powerful generators.

### 3.2. Effect of the Incubation Conditions after PEF Treatment on the Kinetics of Glutathione Extraction from S. cerevisiae

The efficiency of extraction processes depends not only on the treatment applied to facilitate the release of compounds but also on the subsequent extraction conditions. Therefore, to evaluate the impact of extraction conditions, the release of glutathione from electroporated yeast biomass of *S. cerevisiae* treated with PEF (12 kV/cm, 150 µs) for 24 h under various pH and temperature settings was monitored.

**Figure 3:**
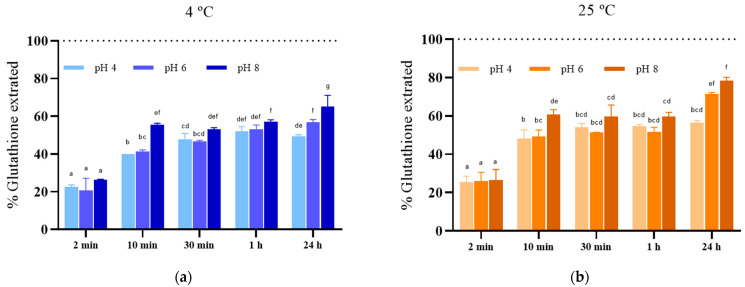
Glutathione extraction yields over time after the PEF treatment (12 kV/cm 150 μs) of *S. cerevisiae* cells and incubated in media with different pH levels (4.0, 6.0, and 8.0) at both 4 °C (**a**) and 25 °C (**b**). Different letters denote significant differences (*p* ≤ 0.05).

Figure 3 illustrates the glutathione extraction yields over time in media with different pH levels (4, 6, and 8) at both 4 °C (Figure 3a) and 25 °C (Figure 3b). After just 2 min of incubation, approximately 20–25% of the total glutathione content in *S. cerevisiae* cells was released, regardless of pH and temperature conditions. Extending the extraction time to 10 min resulted in increased glutathione extraction, reaching levels of 40% and 50% in media with pH 4 and 6, respectively, at both 4 °C and 25 °C. Subsequently, at pH 8, glutathione release was more efficient, achieving extraction yields of 55% at 4 °C and 61% at 25 °C. At 4 °C, extending the incubation period to 24 h did not lead to a significant increase in extraction yield at any pH level. However, after 24 h of incubation, a notable increase in extraction yield was observed in samples incubated at pH 6 and 8, reaching extraction yields of 71% and 78%, respectively. These results align with previous studies, indicating higher extraction yields of intracellular compounds under basic pH conditions in both microalgae [29] and yeast [21].

The rate of release of intracellular compounds from electroporated cells depends, among other factors, on their molecular weight, pore size, and whether the compounds are free in the cytoplasm, bound to other compounds, or located within cellular organelles [19,20,21]. The rapid release of a proportion of glutathione immediately after electroporation, regardless of extraction conditions, suggests the presence of free glutathione in the yeast cytoplasm and indicates that the pore size is sufficient to allow the transport of this molecule through the membrane. These findings are consistent with previous research by Ganeva et al. (2020) [21], who also observed a rapid release of glutathione from electroporated baker’s yeast, achieving an extraction yield of 78 ± 6% within 10 min, with no significant improvement after 4 h of incubation at room temperature.

Glutathione may exist in yeast in various mixed disulfide forms, such as GS-S-CoA, GS-S-Cys, and GS-S-protein, involving reversible attachment to cysteine residues in target proteins [1]. The increased extraction yield observed at 25 °C after 24 h of incubation could be attributed to the hydrolysis of protein-bound glutathione by proteases released from plasmolyzed vacuoles due to osmotic imbalances caused by water entry into the cytoplasm of electroporated yeast cells [25,30]. After the hydrolysis of glutathione bound with high-molecular-weight proteins, this component would become free in the cytoplasm of the cell, enabling it to exit through the cytoplasmic membrane pores. The influence of environmental factors such as pH and temperature on the activity of proteases involved in the release of glutathione attached to proteins could explain the higher extraction yield at higher temperatures and pH levels after 24 h of incubation.

### 3.3. Release of Proteins from S. cerevisiae after PEF Treatment

To assess the advantages of electroporation on yeast cells regarding the purity of the resulting extract, the protein released into the extraction media under the same incubation conditions used for glutathione extraction was examined. Given the high protein content of yeasts (48–60% dry basis), these compounds are the primary impurities in glutathione extraction processes [17], often requiring subsequent purification steps [31].

**Figure 4:**
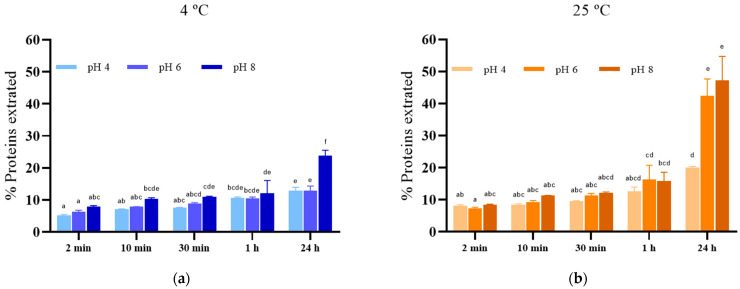
Proteins extracted from electroporated *S. cerevisiae* cells in media with varying pH levels (4.0, 6.0, and 8.0) at 4 °C (**a**) and 25 °C (**b**) over time. Different letters denote significant differences (*p* ≤ 0.05).

Figure 4 illustrates the proteins extracted from electroporated *S. cerevisiae* cells in media with varying pH levels at 4 °C (Figure 4a) and 25 °C (Figure 4b) over time. Similar to glutathione, a rapid release of proteins, representing less than 10% of the total protein content, occurred within the first two minutes of extraction, regardless of extraction conditions. These compounds likely consist of peptides and low-molecular-weight proteins capable of passing through the pores of the cytoplasmic membrane. While there was a modest increase in protein extraction at up to one hour of incubation, a significant increase was observed after 24 h, particularly at 4 °C in pH 8 media and at 25 °C in pH 6 (42%) and 8 (47%) media.

The enhancement of protein extraction from electroporated yeast cells by extending the incubation time has previously been documented [32]. This improvement has been linked to an enlargement of pore size during incubation, facilitating the release of larger molecules [33]. However, prolonged incubation also leads to an increase in the free amino acid concentration in the extraction medium [20,34], indicating proteolysis catalyzed by endogenous proteases in the cytoplasm. This process reduces the protein size, facilitating their passage through the cytoplasmic membrane pores. Consequently, to obtain extracts with minimal low-molecular-weight impurities from electroporated yeast cells, incubation conditions that inhibit proteolytic activity will be necessary, as they will result in a lower protein content in the GSH extract, increasing its purity.

### 3.4. Comparison of PEF with Bead Milling and Hot Water for Extracting Glutathione from S. cerevisiae

In order to assess the feasibility of PEF technology as an alternative to conventional extraction methods for intracellular compounds, the extraction of glutathione from electroporated *S. cerevisiae* cells was compared with bead milling and hot water treatments. Bead milling, a physical procedure, results in complete cell destruction, allowing for immediate recovery of all intracellular compounds. On the other hand, hot water treatment is the conventional method used for industrial-scale glutathione extraction [17]. Although various scientific publications have referenced the utilization of this method, there is uncertainty regarding the precise operational parameters, including temperature ranges of 78–100 °C and extraction durations ranging from 3 to 15 min [1,17,31,35]. For this investigation, treatment conditions (95 °C, 3 min) proposed by [36] were applied to the yeast biomass.

Table 1 compares the amounts of glutathione and proteins extracted per gram of dry biomass for the three procedures tested. Additionally, the increase in the glutathione/protein ratio, serving as an indirect measure of extract purity, is presented. Based on previous findings regarding the influence of pH on glutathione extraction, a pH of 8.0 was chosen for the extraction medium.

The efficacy of bead milling in causing complete cellular destruction was confirmed via optical microscopy, leading to the release of the total intracellular content of glutathione (10.7 ± 0.3 mg/g d_w_) and proteins (637.9 ± 25.7 mg/g d_w_). These concentrations align with ranges typically reported in the literature for *S. cerevisiae*, indicating glutathione concentrations ranging from 0.1 to 1% (d_w_ of yeast) [35] and protein concentrations from 48 to 60% (d_w_ of yeast) [37].

Data presented in Table 1 reveal that PEF and hot water treatments, which did not cause complete cell destruction, were less effective than bead milling for extracting glutathione. While the amount of glutathione extracted by hot water was less than half of the total content in the cells, the PEF treatment permitted extraction of between 59 and 78%. Moreover, these results are not only comparable to, but even superior to, those reported by other authors who utilized 25% ethanol for 60 min for the extraction of glutathione from yeast [17].

In terms of protein extraction, only around 10% of the total protein content was extracted from cells treated by PEF, incubated for 1 h or heat-treated. However, with an increased incubation time for electroporated cells, protein extraction increased by 23% at 4 °C and 47% at 25 °C.

Apart from the extraction yield, the selection of the most suitable extraction procedure depends on various other factors, including extract purity, production costs, environmental impact, and safety considerations [38]. In this study, the increase in the glutathione/protein ratio was used to assess the purity of the glutathione extract obtained from different extraction procedures. According to this parameter, Table 1 indicates that the least protein-contaminated extract was obtained from electroporated cells after 1 h of incubation at 4 °C, being five times purer than the extract obtained by bead milling. Conversely, the purity of the extract from cells treated with hot water was similar to that obtained from electroporated cells after 24 h of incubation at 4 °C.

**Figure 5:**
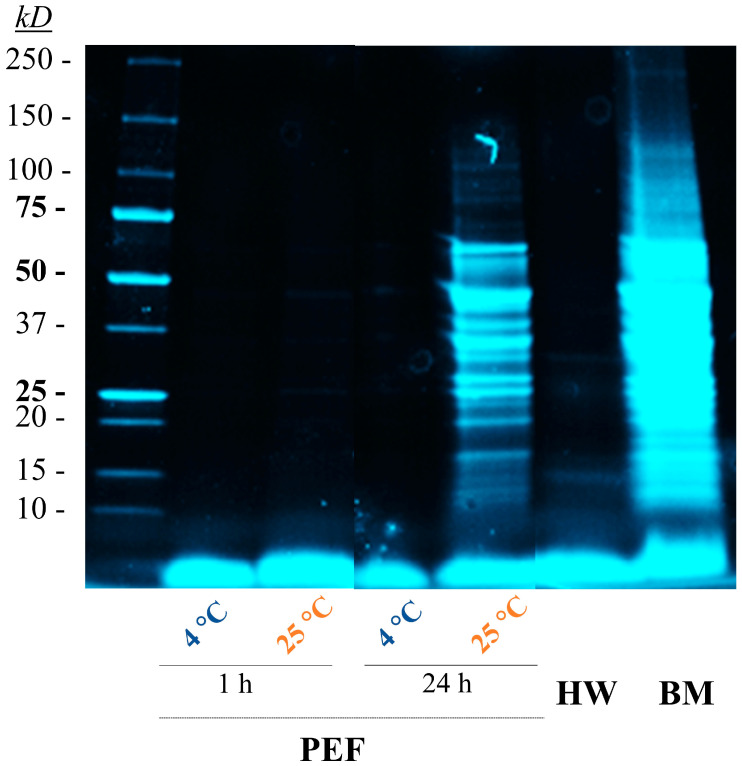
SDS-PAGE analysis of the GSH extracts obtained from *S. cerevisiae* with different extraction methods: hot water (HW), bead milling (BM), and PEF treatments with different incubation times and temperatures. Separation was performed using a 4–15% protein gel. Lane 1: Precision Plus Protein™ Standard (Bio-Rad). The original electrophoresis gel is enclosed in the Supplementary Materials (Figure S1).

Analysis of the SDS-PAGE profile of the extracts (Figure 5) revealed that the extract obtained by bead milling contained proteins with molecular weights up to 150 kDa. In contrast, extracts from cells treated with PEF at 4 °C or 25 °C for 1 h, similar to those treated with hot water, contained proteins and peptides with molecular weights below 10 kDa. Furthermore, extending the incubation time at 25 °C to 24 h resulted in the presence of larger proteins up to 70 kDa in the extract. Considering that the yeast cell wall is permeable to proteins with molecular weights of up to 400 kDa [39,40], these observations support the hypothesis that protein hydrolysis conducted by endogenous proteases released from yeast vacuoles after electroporation facilitates the release of proteins through the electroporated cell membrane. Reducing the incubation temperature and time decreases protease activity and consequently the release of larger protein fragments in the extract.

When comparing PEF technology with hot water in terms of glutathione extraction and extract purity, it was found that electroporation allowed for the extraction of a glutathione-rich extract with a lower protein content after 1 h of extraction at both temperatures tested. Therefore, electroporation of yeast cells could enable the production of tailored glutathione extracts in terms of concentration and purity by adjusting extraction conditions. Meanwhile, decreasing the incubation time and/or temperature could result in purer extracts with lower glutathione concentrations, and increasing the extraction efficiency by extending the incubation time and temperature could lead to less pure extracts.

Furthermore, in terms of energy requirements, although cell electroporation necessitates the application of very high voltages between the electrodes, the short duration of the treatments (µs) means that the treatment demands much less total specific energy (55 kJ/kg) compared to the energy required to increase the temperature from 20 to 95 °C (315 kJ/kg).

### 3.5. Antioxidant Capacity of GSH Extracts

**Figure 6:**
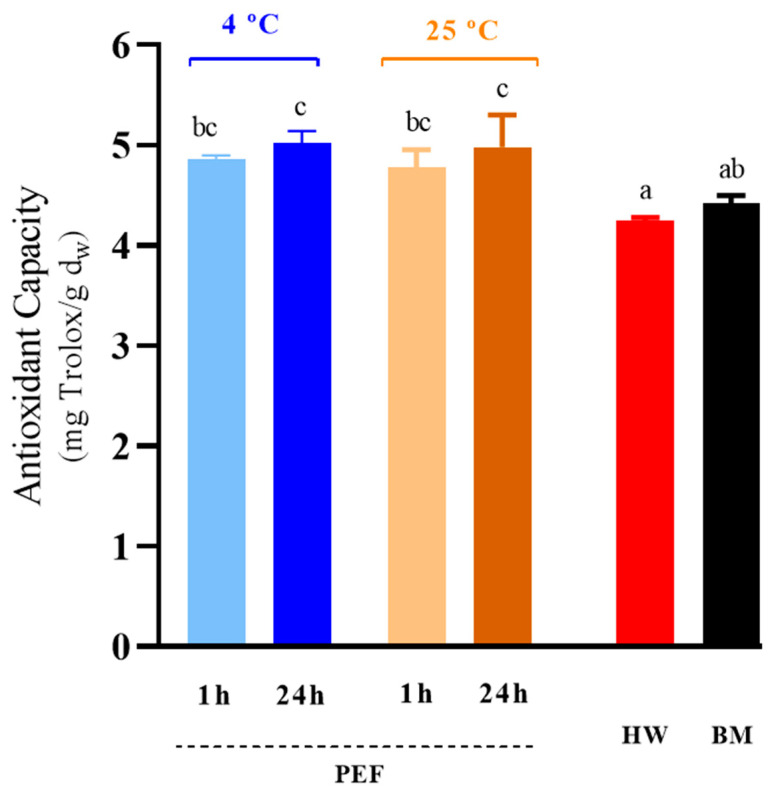
Antioxidant capacity of GSH extracts obtained through PEF, incubated for 1 and 24 h at 4 and 25 °C, together with those obtained via hot water (HW) and bead milling (BM) treatments. Different letters denote significant differences (*p* ≤ 0.05).

The remarkable antioxidant capacity of glutathione makes it a key attribute, particularly significant in the food industry [10,12]. Figure 6 compares the antioxidant capacity of extracts obtained through the procedures analyzed in the preceding section, as determined by DPPH radical scavenging activity. Generally, regardless of incubation conditions, extracts from electroporated cells exhibited a higher antioxidant capacity than those from cells treated by bead milling or hot water, although no statistically significant differences (*p* ≤ 0.05) were found between bead milled extracts and extracts obtained after 1 h of incubation. Santiago and Mori (1993) [41] demonstrated that a significant portion, approximately 85–90%, of observed free radical scavenging activity in the soluble fraction post mechanical cell disruption of yeast is attributable to compounds with molecular weights below 10 kDa, with glutathione widely acknowledged as a primary contributor to the antioxidant capacity of yeast extracts [7].

It would be expected that the extract obtained from cells treated with the bead mill would have the highest antioxidant activity due to the higher glutathione concentration. However, the lower purity of the extract obtained could negatively interfere with the antioxidant properties, resulting in lower-than-expected activity. Conversely, the lower antioxidant activity of the extract obtained by hot water may be associated with its lower glutathione concentration.

## 4. Conclusions

While glutathione holds great potential for various industrial sectors, particularly in the food industry, where it could play a crucial role in preserving product quality, its high cost presents significant challenges for widespread application. Enhancing production methods stands as a viable approach to reducing the price of GSH by lowering production expenses.

This study highlights the potential of PEF technology for the more selective and efficient extraction of glutathione from *S. cerevisiae*. The results underscore the capability of PEF to extract glutathione while minimizing the co-extraction of proteins. Additionally, the extracted glutathione demonstrates a heightened antioxidant capacity, further underlining the effectiveness of PEF in preserving the bioactivity of the compound.

Moreover, the adaptability of extraction conditions post PEF treatment to accommodate diverse requirements has been shown. These findings set the stage for customized optimization of PEF extraction protocols tailored to obtaining specific extracts, enhancing the feasibility of integrating this technology into large-scale production processes. Future investigations on the potential for scaling up glutathione extraction using PEF from glutathione-overproducing yeast strains, and evaluating their properties in various food matrices, are warranted.

## Figures and Tables

**Table 1:** Comparison of glutathione and protein concentrations of extracts obtained by PEF, incubated for 1 and 24 h at 4 and 25 °C, with hot water (HW) and bead milling (BM). The ratio Δ glutathione/protein normalized to the ratio obtained by bead milling of the extracts is also shown. Different letters denote significant differences (*p* ≤ 0.05).

Extraction Method			Glutathione Concentration (mg/g d_w_)	Protein Concentration (mg/g d_w_)	Δ Ratio Glutathione/Proteins
**Bead mill** (BM)			10.7 ± 0.3 *d*	637.9 ± 25.7 *d*	1.0 *a*
**PEF** (12 kV/cm, 150 µs)	4 °C	1 h	6.3 ± 0.2 *b*	77.4 ± 18.3 *a*	5.2 ± 1.4 *d*
		24 h	6.9 ± 0.5 *b*	152.2 ± 7.5 *b*	2.7 ± 0.3 *bc*
	25 °C	1 h	6.4 ± 0.2 *b*	101.1 ± 10.9 *a*	3.8 ± 0.5 *cd*
		24 h	8.4 ± 0.1 *c*	304.4 ± 24.6 *c*	1.6 ± 0.2 *ab*
**Hot water** (HW) (95 °C, 3 min)			4.9 ± 0.1 *a*	78.5 ± 4.9 *a*	3.7 ± 0.3 *c*

## Data Availability

The original contributions presented in the study are included in the article, further inquiries can be directed to the corresponding author.

## Supplementary Materials

The following supporting information can be downloaded at: https://www.mdpi.com/article/10.3390/foods13121916/s1, Figure S1: Original SDS-PAGE gel of the GSH extracts obtained from *S. cerevisiae* with different extraction methods; hot water (HW), bead mill (BM), ethanol (etOH) and PEF treatments with different incubation times and temperature. Separation was performed using a 4–15% protein gel. Lane 1: Precision Plus Protein™ Standard (Bio-Rad).
