# Supplementary material for: Pulsed Electric Field Technology for the Extraction of Glutathione from Saccharomyces cerevisiae
Source: Foods. 2024 Jun 18;13(12):1916. doi: 10.3390/foods13121916 (PMC11203235; doi:10.3390/foods13121916)
Supplement: Supplementary file 1 [file foods-13-01916-s001.zip › foods-3024163-supplementary.PDF]

Supplementary

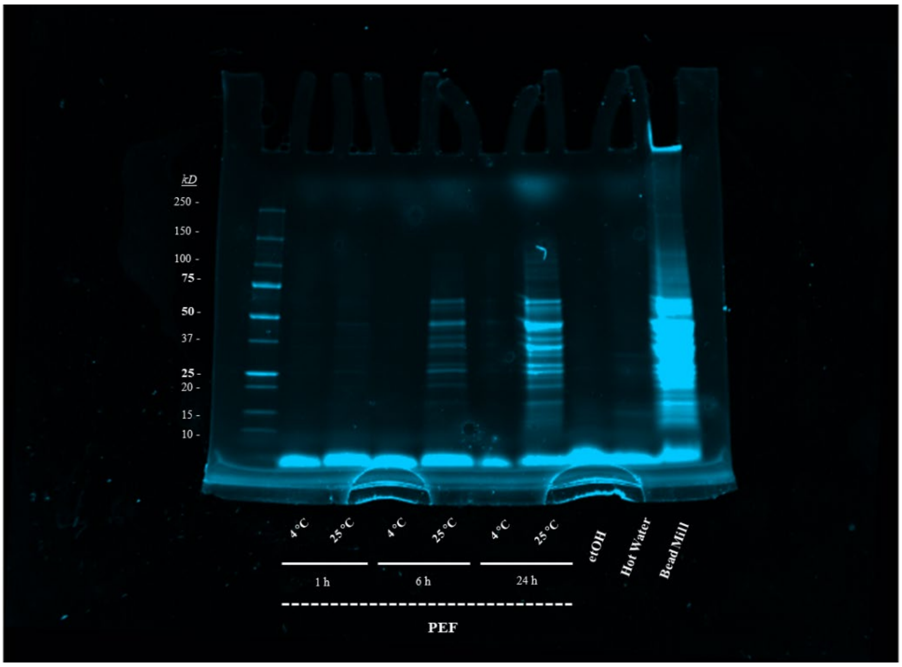

Suppl. Figure 1: Original SDS-PAGE gel of the GSH extracts obtained from *S. cerevisiae* with different extraction methods; hot water (HW), bead mill (BM), ethanol (etOH) and PEF treatments with different incubation times and temperature. Separation was performed using a 4-15% protein gel. Lane 1: Precision Plus Protein™ Standard (Bio-Rad).
